# HIV-1 tropism in low-level viral load HIV-1 infections during HAART in Guangdong, China

**DOI:** 10.3389/fmicb.2023.1159763

**Published:** 2023-04-20

**Authors:** Chuyu Zhang, Yun Lan, Linghua Li, Ruiying He, Yu Meng, Jian Li, Weilie Chen

**Affiliations:** ^1^Institute of Infectious Diseases, Guangzhou Eighth People’s Hospital, Guangzhou Medical University, Guangzhou, Guangdong, China; ^2^Infectious Disease Center, Guangzhou Eighth People’s Hospital, Guangzhou Medical University, Guangzhou, Guangdong, China

**Keywords:** HIV-1, HIV-1 tropism, low-level viral load, HIV-1 V3 loop, CRF07_BC, CRF01_AE

## Abstract

**Background:**

Since only a few studies have been conducted on the factors associated with different HIV-1 tropisms in low-level viral load HIV-1 infections in China, we investigated the sequences of HIV-1 V3 loop in prevalent HIV-1 subtypes and factors related to HIV-1 tropism and immune recovery in HIV-1 infections after 6 months of highly active antiretroviral therapy (HAART) in Guangdong, China.

**Methods:**

Plasma samples with HIV-1 RNA of 400–999 copies/mL were collected. We analyzed the amino acid sequence of the V3 loop by *in silico* prediction algorithms. Mann–Whitney and Chi-square tests were used for statistical comparison. Furthermore, logistic regression and multiple linear regression were used, respectively, for factors associated with 351 HIV-1 tropism and immune recovery of 67 cases with continued CD4^+^ T cell count during HAART.

**Results:**

There was a lower percentage of HIV-1 R5-tropic virus in CRF01_AE (66.3%) (*p* < 0.0001) and CRF55_01B (52.6%) (*p* < 0.0001) compared with both CRF07_BC (96.1%) and CRF08_BC (97.4%), respectively. Compared with the R5-tropic virus, higher proportions of IIe8/Val8, Arg11/Lys11, and Arg18/His18/Lys18 were observed in the X4-tropic virus of CRF01_AE and CRF07_BC (*p* < 0.0001). The baseline CD4^+^ T cell count (*p* < 0.0001) and baseline CD4^+^ T/CD8^+^ T ratio (*p* = 0.0006) of all R5-tropic infections were higher than those in the X4-tropic infection. The baseline CD4^+^ T cell count (odds ratio [OR] 0.9963, *p* = 0.0097), CRF07_BC (OR 0.1283, *p* = 0.0002), and CRF08_BC (OR 0.1124, *p* = 0.0381) were associated with less HIV-1 X4-tropism. The baseline CD4^+^ T cell count was a positive factor (*p* < 0.0001) in the recovery of CD4^+^ T cell count during HAART.

**Conclusion:**

R5-tropism represented the majority in low-level viral load HIV-1 infections receiving HAART for more than 6 months in Guangdong, China. The baseline immune level in the HIV-1 R5-tropic infections was higher than that in the X4-tropic infections. The amino acids of the 8th, 11th, and 18th of the HIV-1 V3 loop were more variable in the X4-tropic HIV-1. CRF01_AE, CRF55_01B, and lower baseline CD4^+^ T cell count were associated with more HIV-1 X4-tropism. The immune recovery during HAART was positively related to baseline CD4^+^ T cell count.

## 1. Introduction

Associated with the severity of illness, the human immunodeficiency virus 1 (HIV-1) has a phenotype with preferential binding to CCR5 (C-C motif receptor 5) or CXCR4 (C-X-C motif receptor 4) coreceptors, which is called HIV-1 tropism (Berger et al., 1998; Tuttle et al., 1998; Yi et al., 1999). HIV-1 V3 loop, the 3rd high variable region of HIV-1 gp120, is a semi-conserved structure that changes its space position after the connection of gp120 and receptor CD4 (Huang et al., 2007; Xiang et al., 2010; Tan et al., 2013). The amino acid and charges of the HIV-1 V3 loop result in the HIV-1 preferences for coreceptors CCR5 or CXCR4 (Briggs et al., 2000; Schuitemaker et al., 2011). Compared with R5-tropic HIV-1, X4-tropic HIV-1 relates to HIV-1 reservoir (Delobel et al., 2005; Abbate et al., 2011; Zhang et al., 2013; Roche et al., 2020) and lower immune recovery (Connor et al., 1997; Ge et al., 2021) in HIV-1 infected individuals receiving highly active antiretroviral therapy (HAART). *In silico* prediction based on the nucleotide sequence of HIV-1 gp120 V3 loop can be used as a surveillance tool for HIV-1 tropism and the resistance against CCR5 inhibitors prior to phenotypic detection (Lengauer et al., 2007; Bunnik et al., 2011; Swenson et al., 2011; Panos and Watson, 2015; Zhou et al., 2016). However, it is not fully clear what factors are associated with the V3 loop and HIV-1 tropism (Saracino et al., 2009), while there are distinguishing proportions of different HIV-1 tropisms in different HIV-1 clades and clusters (Song et al., 2019; Ge et al., 2021).

Despite receiving HAART, the HIV-1 reservoir results in HIV-1 RNA rebound, reaching a detectable level (Bednar et al., 2016; Aamer et al., 2020; Margolis et al., 2020). Indeed, intermittent low-level viremia (“blip”) and persistent low-level viremia (pLLV) have been commonly observed in treated persons living with HIV (PLWH) (Nettles et al., 2005; Crespo-Bermejo et al., 2021). Previous investigations have revealed that persistent and higher low-level viremia positively relates to virologic failure (Joya et al., 2019; Li et al., 2021) and further disease progression, including acquired immunodeficiency syndrome (AIDS) events and non-AIDS-defining events (NADEs) (Neuhaus et al., 2010; Wong et al., 2018; Elvstam et al., 2021). The relationship between HIV-1 RNA rebound and HIV-1 tropism is still unclear. Additionally, little research has focused on the HIV-1 V3 loop and tropism in Chinese treated persons living with low-level HIV-1 viremia and receiving HAART.

Considering the above-stated, it makes sense to examine the correlation between HIV-1 tropism and clinical characteristics in various HIV-1 subtypes and virologic responses. We retrospectively investigated the characteristics of the V3 loop and factors associated with HIV-1 tropism in low-level viral load HIV-1 infections in Guangdong, China.

## 2. Materials and methods

### 2.1. Study design

**Figure 1:**
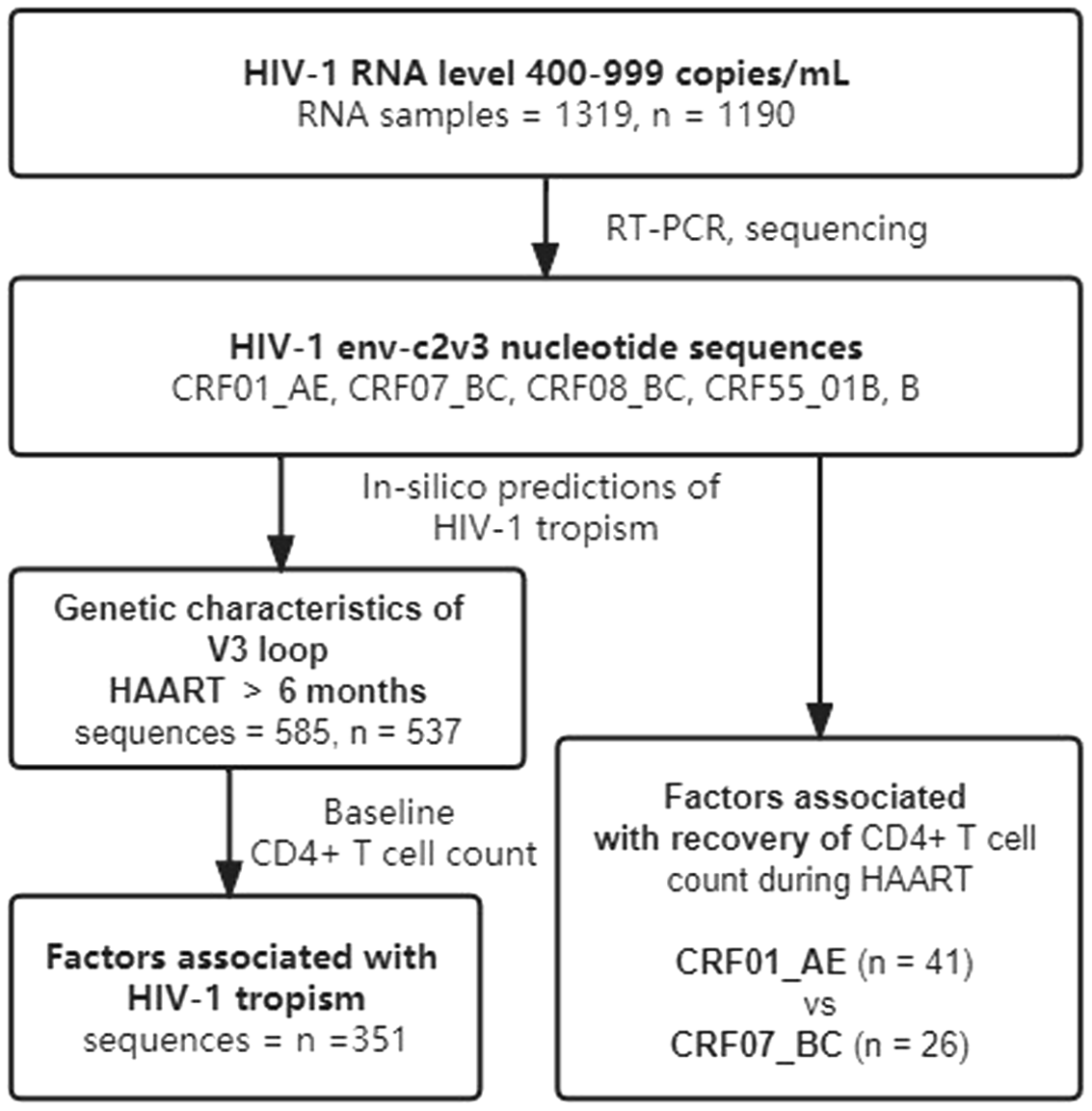
Diagram of the study design. HAART, highly active antiretroviral therapy.

A total of 1,319 plasma samples with low-level HIV-1 RNA load (400–999 copies/mL) were collected from 1,190 HIV-1 infections receiving HAART from 2014 to 2020 (Figure 1) in Guangdong, China. We enrolled these samples together with information on their HIV-1 genotype and demographic characteristics from the Guangdong AIDS Diagnosis and Treatment Quality Control Center. Excluding the critical missing data and cases failed in genotyping and sequencing, statistical analysis of demographic characteristics and clinical laboratory tests were finally conducted in 537 cases. For factors associated with HIV-1 tropism, 351 HIV-1 infections were eligible because they had baseline CD4^+^ T cell count (baseline CD4^+^ T cell count was defined as the CD4^+^ T cell count within 31 days before HAART initiation) and received HAART over 6 months (Table 1). A total of 67 HIV-1 infections with continued CD4^+^ T cell count during HAART were enrolled to analyze factors associated with immune recovery. Furthermore, we specifically analyzed 6 cases of HIV-1 tropism switch (Supplementary Table 3).

**Table 1:** Demographics and characteristics of the cases with baseline CD4^+^ T cell count (*N* = 351).

Variable	Overall (*n* = 351)	HIV-1 subtypes					*p*-Value
		CRF01_AE (*n* = 210)	CRF07_BC (*n* = 88)	CRF08_BC (*n* = 23)	CRF55_01B (*n* = 17)	B (*n* = 13)	
Age, median (IQR), y	42 (35, 52)	43 (34.75, 55)	43 (35, 50.75)	40 (35, 45)	37 (31.5, 45.5)	42 (33, 46)	0.3364
Gender, *n* (%)							0.1042
Male	264 (75.2)	155 (73.8)	66 (75.0)	15 (65.2)	17 (100.0)	11 (84.6)	
Female	87 (24.8)	55 (26.2)	22 (25.0)	8 (34.8)	0	2 (15.4)	
Delay of HAART initiation, median (IQR), *d*	36 (17, 406)	40.5 (18, 336)	35 (15, 864.75)	43 (24, 572)	21 (13, 40)	18 (7.5, 887.5)	0.2646
Time on HAART, median (IQR), d	1,036 (529, 1,797)	1,123.5 (674.5, 1,987.25)	866.5 (409.75, 1,486.25)	1,024 (551, 2,568)	448 (313.5, 1,037)	898 (416, 1,388)	0.0011
Plasma HIV-1 RNA (copies/mL), median (IQR)	624 (493, 774)	600 (484, 758.5)	671.5 (537.25, 812)	678 (485, 721)	683 (528.5, 844.5)	605 (479.5, 761)	0.4477
Baseline CD4+ T cell count, median (IQR)	181 (61, 281)	145 (29, 232.75)	252.5 (156, 373.75)	262 (185, 359)	193 (52.5, 343.5)	174 (36, 223.5)	<0.0001
Baseline CD8+ T cell count, median (IQR)	686 (370, 1,116)	659.5 (348.5, 960.25)	802 (415.75, 1,243.25)	671 (0, 1,169)	959 (691.5, 1339.5)	585 (269, 922)	0.0323
Baseline CD4+/CD8+ T cell count, median (IQR)	0.17 (0.0775, 0.32)	0.14 (0.05, 0.2575)	0.255 (0.13, 0.3725)	0.27 (0.15, 0.3975)	0.145 (0.0475, 0.385)	0.195 (0.13, 0.2725)	<0.0001
In-silico predictions of HIV-1 co-receptor usage, *n* (%)							<0.0001
CCR5-using	263 (74.9)	138 (65.7)	84 (95.5)	22 (95.7)	8 (47.1)	11 (84.6)	
CXCR4-using	88 (25.1)	72 (34.3)	4 (4.5)	1 (4.3)	9 (52.9)	2 (15.4)	

Data are presented as no. (%).

HAART, Highly active antiretroviral therapy; NRTIs, Nucleoside reverse transcriptase inhibitors; NNRTIs, Non-nucleoside reverse transcriptase inhibitors.

### 2.2. Viral RNA extraction, nested reverse-transcription polymerase chain reaction amplification, and sequencing

HIV-1 RNA was extracted from plasma samples using the TIANamp Virus RNA Kit (TIANGEN) following the manufacturer’s protocol. The primers of HIV-1 env-c2v3 region for nested reverse-transcription polymerase chain reaction (RT-PCR) were composed of out forward (P5-1-S) 5’-GTACACATGGAATTAAACCAGT-3′ (nt 6,964–6,985), out reverse (PV3) 5’-CAGTAGAAAAATTCCCCTCCACAATTAA-3′ (nt 7,351–7,378), inner forward (DR7-S) 5’-TCAACTCAACTGCTGTTAAATGG-3′ (nt 6,990–7,012), and inner reverse (BSUI2) 5’-TTRYAATTTCTRGRTCCCCTCC-3′ (nt 7,320-7,341). The first round of PCR using primer P5-1-S and PV3 was conducted by the PrimeScript™ One-Step RT-PCR Kit Ver.2 (Takara) with the following conditions: 50°C for 30 min, 94°C for 2 min, 35 cycles of 94°C for 30s, 55°C for 30s, 72°C for 1 min and followed by 72°C for 10 min. The second round of PCR using DR7-S and BSUI2 was conducted by the Premix Taq™ (Takara) with the following conditions: 94°C for 2 min, 40 cycles of 94°C for 30s, 55°C for 30s, 72°C for 40s and followed by 72°C for 10 min. The products of PCR were sequenced using the inner primers on an automated sequencer (ABI 3730xL) after purification.

### 2.3. Viral tropism prediction

The nucleotide sequences of HIV-1 gp120 env-c2v3 region were edited with Sequencher 5.4.6 (USA). All eligible sequences were converted to about 35 amino acids of the V3 loop, and coreceptor usages were conducted with *in silico* prediction methods, including the Geno2pheno algorithm (with a false-positive rate cutoff value estimated at 10% for X4-tropism),^1^ PhenoSeq,^2^ and HIVcoPRED.^3^ The final predictions of coreceptor usages, CCR5-using or CXCR4-using, were judged on the results from the above-mentioned online methods.

### 2.4. Phylogeny analysis

Phylogeny trees were constructed by neighbor-joining and 1,000 bootstraps in Kimura 2-parameter model in MEGA version 11.0.13. Distance estimations of sequences env-c2v3 were calculated using 1,000 bootstraps and the Kimura 2-parameter model in MEGA version 11.0.13.

### 2.5. Statistical analysis

Statistical analysis was performed using GraphPad Prism 9, and *p* < 0.05 was considered statistically significant. Kruskal-Wallis and Mann–Whitney tests were used to compare age, delay of HAART initiation, duration of HAART, HIV-1 RNA load, baseline CD4^+^ T cell count, and baseline CD4^+^ T/CD8^+^ T ratio. Gender, initially prescribed HAART regimen, route of infection, and HIV-1 tropism were analyzed by Fisher’s exact and Chi-square tests. Univariate and multivariate logistic regressions were chosen for the analysis of factors associated with HIV-1 tropism. Factors associated with immune recovery were analyzed by multiple linear regression. Statistical comparison of sequences env-c2v3 distance estimates was performed by ordinary one-way ANOVA. The LOWESS curve was used to graph the CD4^+^ T cell trajectories during HAART based on HIV-1 CRF01_AE and CRF07_BC.

## 3. Results

### 3.1. Characteristics of the study population

Excluding the critical missing data and cases failed in genotyping and sequencing, we obtained 585 env-c2v3 sequences and enrolled 537 individuals. Of 537 PLWH who received HAART for more than 6 months (Table 2), there were 312 individuals with CRF01_AE (58.1%), 153 individuals with CRF07_BC (28.5%), 38 individuals with CRF08_BC (7.1%), 19 individuals with CRF55_01B (3.5%), and 15 individuals with subtype B (2.8%). The delay of HAART initiation in CRF07_BC was longer (*p* = 0.0006). The time on HAART in CRF55_01B was shorter (*p* < 0.0001). Among five HIV-1 subtypes, no significant difference was observed in the comparing of the age (*p* = 0.4200), gender (*p* = 0.0899), and initial HAART regimen prescribed (*p* = 0.1576). Notably, plasma HIV-1 RNA nearly displayed a statistical difference (*p* = 0.0527).

**Table 2:** Demographics and characteristics of the study population (*N* = 537).

Variable	Overall (*n* = 537)	HIV-1 subtypes					*p*-Value
		CRF01_AE (*n* = 312)	CRF07_BC (*n* = 153)	CRF08_BC (*n* = 38)	CRF55_01B (*n* = 19)	B (*n* = 15)	
Age, median (IQR), y	42 (35, 50.5)	42 (35.25, 53)	43 (35, 49)	40 (34, 47)	39 (32, 49)	42 (37, 47)	0.4200
Gender, *n* (%)							0.0899
Male	414 (77.1)	233 (74.7)	121 (79.1)	28 (73.7)	19 (100.0)	13 (86.7)	
Female	123 (22.9)	79 (25.3)	32 (20.9)	10 (26.3)	0	2 (13.3)	
Delay of HAART initiation, median (IQR), d	77 (23, 706)	68 (23.25, 489)	239 (26.5, 1,464)	93 (33.75, 251.75)	23 (15, 72)	50 (8,953)	0.0006
Time on HAART, median (IQR), d	1,017 (496, 1,777.5)	1122.5 (620.5, 1,995)	835 (431.5, 1,480.5)	854 (295.5, 2,028.25)	448 (321, 1,036)	898 (440, 1,474)	<0.0001
Plasma HIV-1 RNA (copies/mL), median (IQR)	642 (508.5, 782.5)	614.5 (492, 765.5)	679 (540.25, 836.5)	651.5 (480.5, 741.75)	683 (588, 837)	549 (454, 726)	0.0527
Initial HAART regimen prescribed, n (%)							0.1576
NRTIs + NNRTIs	475 (88.5)	268 (85.9)	141 (92.2)	34 (89.5)	19 (100.0)	13 (86.7)	
Others	62 (11.5)	44 (14.1)	12 (7.8)	4 (10.5)	0	2 (13.3)	
Routes of infection, *n* (%)							0.0012
Sexual transmission	296 (55.1)	180 (57.7)	73 (47.7)	17 (44.7)	15 (78.9)	11 (73.3)	
Non sexual transmission	200 (37.2)	108 (34.6)	71 (46.4)	19 (50.0)	1 (5.3)	1 (6.7)	
N/A	41 (7.6)	24 (7.7)	9 (5.9)	2 (5.3)	3 (15.8)	3 (20.0)	
In-silico predictions of HIV-1 co-receptor usage, *n* (%)							<0.0001
CCR5-using	413 (76.9)	207 (66.3)	147 (96.1)	37 (97.4)	10 (52.6)	12 (80.0)	
CXCR4-using	124 (23.1)	105 (33.7)	6 (3.9)	1 (2.6)	9 (47.4)	3 (20.0)	

Data are presented as no. (%).

HAART, Highly active antiretroviral therapy; NRTIs, Nucleoside reverse transcriptase inhibitors; NNRTIs, Non-nucleoside reverse transcriptase inhibitors.

### 3.2. HIV-1 tropism prediction

**Figure 2:**
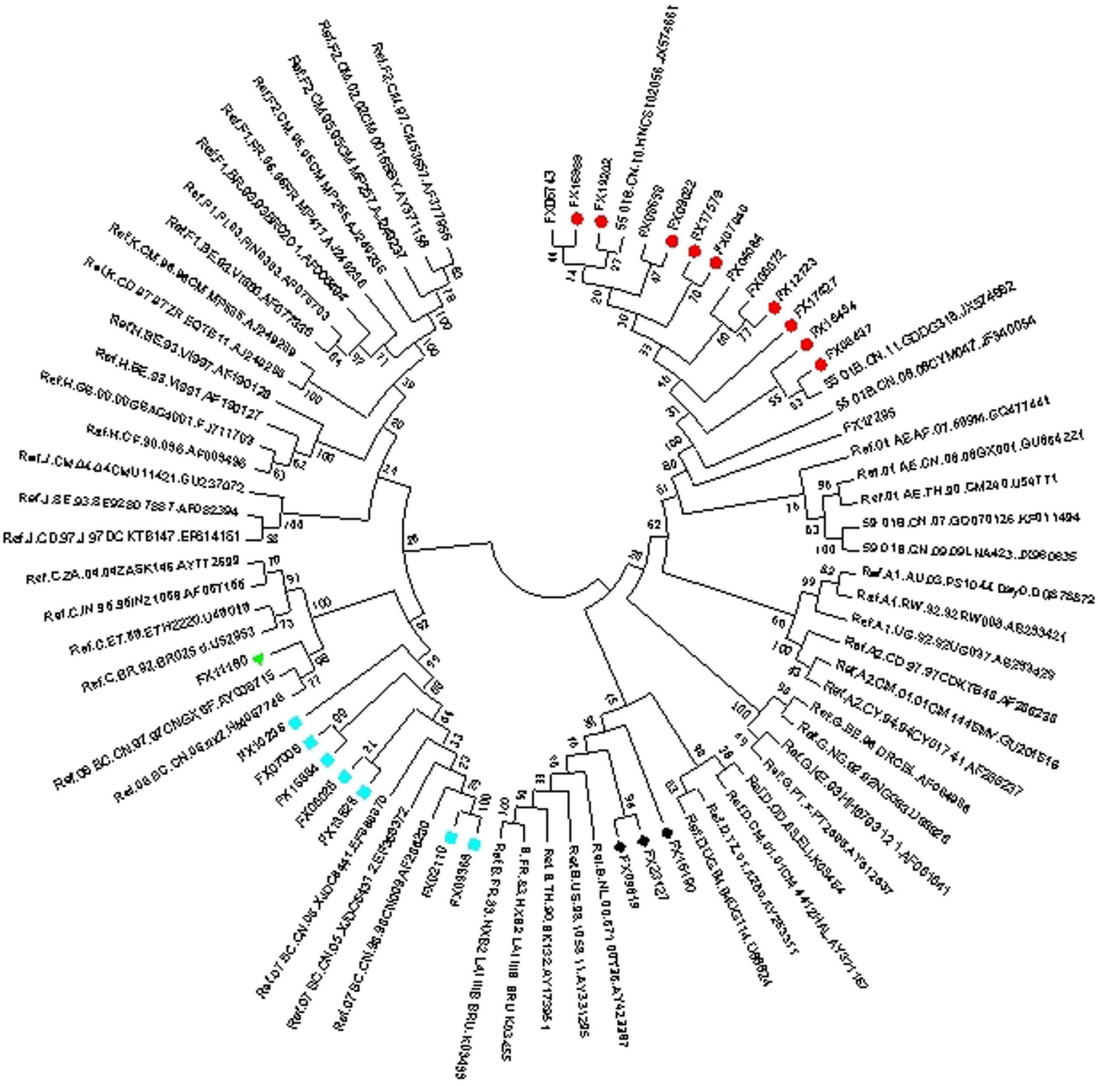
Phylogenetic tree (for HIV-1 subtype confirming) of X4-tropism in CRF07_BC (the cyan square), CRF08_BC (the green triangle), CRF55_01B (the red circle), and subtype B (the black rhombus).

Of 585 env-c2v3 sequences from 537 PLWH who received HAART for more than 6 months, HIV-1 X4-tropism was found in CRF01_AE (X4 33.7%, R5 66.3%, *n* = 312), CRF07_BC (X4 3.9%, R5 96.1%, *n* = 153), CRF08_BC (X4 2.6%, R5 97.4%, *n* = 38), CRF55_01B (X4 47.4%, R5 52.6%, *n* = 19), and subtype B (X4 20.0%, R5 80%, *n* = 15). The proportion of X4-tropism in CRF01_AE and CRF55_01B was higher than that in CRF07_BC (*p* < 0.0001) and CRF08_BC (p < 0.0001). Besides, the X4-tropic virus in subtype B was more commonly represented than in CRF07_BC (*p* = 0.0351). Except for CRF01_AE, the rest of the X4-tropic viruses were represented by a phylogenetic tree (for HIV-1 subtype confirming) (Figure 2), indicating that the genotyping of minority X4-tropic viruses in CRF07_BC, CRF08_BC, CRF55_01B, and subtype B was faultless. The phylogenetic tree was displayed as X4-CRF07_BC (the cyan square), X4-CRF08_BC (the green triangle), X4-CRF55_01B (the red circle), and X4-subtype B (the black rhombus).

### 3.3. Amino acids of HIV-1 Gp120 V3 loop in different HIV-1 subtypes and tropism

Compared with Thr8, Ser11, and Gln18 in the R5-tropic virus, higher proportions of IIe8/Val8, Arg11/Lys11, and Arg18/His18/Lys18 were observed in the X4-tropic virus both of CRF01_AE and CRF07_BC (*p* < 0.0001) (Tables 3, 4). Asp25 (30.7%) and Glu25 (42.1%) were statistically different (*p* = 0.0093) in X4-tropic-CRF01_AE, compared with R5-tropic-CRF01 AE which displayed Asp25 (55.9%) and Glu25 (38.3%). However, Asp25 (53.9%) and Glu25 (25.7%) were not statistically different (*p* = 0.3329) in R5-tropic-CRF07_BC, compared with X4-tropic-CRF07_BC which displayed Asp25 (28.5%) and Glu25 (42.8%). The proportions of amino acids were showed by forms of heat map. We used a gradient ramp to display the percentage of amino acids. The lower the percentage of amino acid, the lighter the color. Of all HIV-1 subtypes (Supplementary Tables 1A–C), we found a rich diversity in the V3 loop with a conserved amino acid as “CTRPXNNTRXSXXXGPGXXFYXTGXIIGDIRXAXC,” while the “X” indicated the heterogeneous amino acids.

**Table 3:** The composition of amino acids in CRF01_AE HIV-1 gp120 V3 loop.

The composition of amino acids in CRF01_AE HIV-1 gp120 V3 loop / %																																								
CCR5-using (sequences = 227)																	Amino acid			CXCR4-using (sequences = 114)																				
																100.0	C	1	C	100.0																				
												0.9	S	7.0	I	92.1	T	2	T	95.6	I	4.4																		
																100.0	R	3	R	99.1	K	0.9																		
														0.4	L	99.6	P	4	P	100.0																				
				0.9	Y	0.9	G	1.3	D	1.3	A	3.5	N	4.4	F	87.7	S	5	S	67.5	F	27.2	Y	2.6	G	1.8	D	0.9												
												1.3	D	1.8	T	96.9	N	6	N	77.1	D	7.9	K	7.0	T	2.6	E	1.8	R	1.8	G	0.9	I	0.9						
																100.0	N	7	N	82.4	K	9.6	Y	4.4	I	1.8	H	0.9	S	0.9										
								0.4	R	0.4	Q	1.3	I	2.2	K	95.7	T	8	T	52.5	I	22.8	V	13.2	K	5.3	R	2.6	A	0.9	F	0.9	M	0.9	Q	0.9				
														2.2	I	97.8	R	9	R	92.9	K	5.3	E	1.8																
				0.4	R	0.4	I	0.9	V	0.9	Q	0.9	E	7.9	K	88.6	T	10	T	59.6	K	16.7	I	7.0	A	4.4	Q	3.5	R	3.5	V	2.6	E	1.8	S	0.9				
														10.6	G	89.4	S	11	S	57.9	R	33.3	K	5.3	G	3.5														
				0.4	Q	0.4	A	1.3	L	13.2	T	13.7	V	19.4	M	51.6	I	12	M	32.4	T	25.4	I	19.3	V	14.9	S	2.6	A	1.8	F	1.8	L	1.8						
		0.4	I	2.6	A	4.0	N	4.8	S	15.0	P	15.0	H	19.4	R	38.8	T	13	T	45.5	R	36.8	P	5.3	H	4.4	S	4.4	A	1.8	F	0.9	N	0.9						
						0.4	T	0.4	F	0.9	L	1.8	V	7.9	M	88.6	I	14	I	65.6	M	23.7	R	2.6	F	1.8	L	1.8	T	1.8	A	0.9	K	0.9	V	0.9				
														1.3	A	98.7	G	15	G	100.0																				
																100.0	P	16	P	97.4	L	2.6																		
												0.4	L	0.4	A	99.2	G	17	G	99.1	A	0.9																		
		0.4	S	0.4	L	0.4	I	0.9	M	1.8	H	2.6	K	10.1	R	83.4	Q	18	Q	45.5	R	28.1	H	16.7	K	8.8	T	0.9												
				1.3	S	2.2	L	2.6	I	4.4	T	5.7	M	10.6	A	73.2	V	19	V	85.1	A	6.1	I	2.6	M	2.6	P	1.8	R	0.9	T	0.9								
						0.4	V	0.9	I	1.3	Y	2.2	W	5.7	L	89.5	F	20	F	78.8	Y	13.2	W	2.6	L	1.8	V	1.8	C	0.9	I	0.9								
														4.0	F	96.0	Y	21	Y	92.9	F	5.3	H	1.8																
				0.4	T	0.9	S	0.9	A	1.8	G	4.0	Q	11.9	K	80.1	R	22	R	58.8	K	22.8	S	14.0	G	2.6	Q	0.9	T	0.9										
		0.4	S	0.4	R	0.4	M	0.4	E	0.4	A	0.9	I	1.8	P	95.3	T	23	T	94.6	I	1.8	Q	1.8	R	1.8														
														1.8	E	98.2	G	24	G	87.7	E	7.0	D	3.5	K	0.9	N	0.9												
				0.4	S	0.9	Q	0.9	N	0.9	G	2.6	A	38.3	E	56.0	D	25	E	42.0	D	30.7	S	7.9	G	7.0	A	4.4	N	4.4	K	1.8	Q	0.9	T	0.9				
												0.4	L	3.5	V	96.1	I	26	I	92.1	M	4.4	V	2.6	L	0.9														
				0.4	S	0.4	L	0.4	A	0.9	K	4.0	V	13.7	T	80.2	I	27	I	55.1	T	24.6	V	7.0	L	3.5	R	2.6	A	1.8	S	1.8	E	0.9	G	0.9	M	0.9	Q	0.9
																100.0	G	28	G	100.0																				
														11.5	N	88.5	D	29	D	94.7	N	5.3																		
												0.4	T	0.4	P	99.2	I	30	I	98.2	P	0.9	T	0.9																
														0.9	K	99.1	R	31	R	97.3	K	1.8	W	0.9																
0.4	L	0.4	G	0.4	E	1.3	N	2.2	S	4.4	A	9.7	R	26.0	Q	55.2	K	32	K	77.2	R	14.9	Q	7.0	N	0.9														
														0.4	V	99.6	A	33	A	100.0																				
				0.4	W	0.4	S	0.4	Q	0.4	G	6.2	H	8.8	F	83.4	Y	34	Y	81.5	H	9.6	F	4.4	N	1.8	A	0.9	Q	0.9	R	0.9								
																100.0	C	35	C	100.0																				

A, alanine; C, cysteine; D, aspartic acid; E, glutamic acid; F, phenylalanine; G, glycine; H, histidine; I, isoleucine; K, lysine; L, leucine; M, methionine; N, asparagine; P, proline; Q, glutamine; R, arginine; S, serine; T, threonine; V, valine; W, tryptophan; Y, tyrosine. The colors indicate different percentage of amino acids. The lower the percentage of amino acid, lighter the color.

**Table 4:** The composition of amino acids in CRF07_BC HIV-1 gp120 V3 loop.

The composition of amino acids in CRF07_BC HIV-1 gp120 V3 loop / %																														
CCR5-using (sequences = 163)																			Amino acid			CXCR4-using (sequences = 7)								
																		100.0	C	1	C	100.0								
										0.6	L	1.2	V	1.8	A	11.7	I	84.7	T	2	T	85.7	A	14.3						
																0.6	G	99.4	R	3	R	100.0								
														1.2	L	1.8	T	97.0	P	4	P	100.0								
								0.6	C	1.2	D	1.2	A	8.0	S	36.8	G	52.2	N	5	S	85.7	G	14.3						
																		100.0	N	6	N	85.7	D	14.3						
																		100.0	N	7	N	100.0								
																0.6	A	99.4	T	8	T	85.7	I	14.3						
																0.6	S	99.4	R	9	R	100.0								
										2.5	E	3.7	Q	5.5	T	6.7	R	81.6	K	10	T	42.9	K	42.8	Q	14.3				
																1.8	G	98.2	S	11	S	57.1	R	28.6	G	14.3				
												1.2	M	1.8	T	14.7	V	82.3	I	12	V	42.8	I	28.6	T	28.6				
										0.6	S	0.6	P	1.2	H	2.5	G	95.1	R	13	T	28.6	R	28.6	A	14.3	G	14.3	H	14.3
																1.2	M	98.8	I	14	I	71.4	M	28.6						
																		100.0	G	15	G	100.0								
																		100.0	P	16	P	100.0								
																		100.0	G	17	G	100.0								
														0.6	K	0.6	R	98.8	Q	18	Q	71.4	R	28.6						
								1.2	S	1.2	M	1.2	I	1.8	V	10.4	A	84.2	T	19	V	71.4	A	14.3	T	14.3				
														1.2	L	4.3	I	94.5	F	20	F	71.4	I	28.6						
																3.7	F	96.3	Y	21	Y	100.0								
														1.2	T	2.5	R	96.3	A	22	R	28.6	A	28.6	K	28.5	S	14.3		
0.6	S	0.6	Q	0.6	P	0.6	I	0.6	A	1.2	R	1.2	H	1.8	Y	1.8	M	91.0	T	23	T	85.7	R	14.3						
														0.6	E	0.6	K	98.8	G	24	G	85.7	K	14.3						
		0.6	V	0.6	T	0.6	S	0.6	R	3.7	Q	5.5	A	8.6	G	25.8	E	54.0	D	25	E	42.9	D	28.6	A	14.3	Q	14.3		
																7.4	V	92.6	I	26	I	100.0								
														1.8	V	3.1	T	95.1	I	27	I	85.7	V	14.3						
																		100.0	G	28	G	100.0								
														0.6	E	16.0	N	83.4	D	29	D	100.0								
																1.2	P	98.8	I	30	I	100.0								
																0.6	K	99.4	R	31	R	100.0								
0.6	Y	0.6	S	0.6	G	1.2	N	1.2	H	1.8	L	3.7	E	5.5	K	6.7	R	78.1	Q	32	K	57.1	Q	28.6	N	14.3				
																		100.0	A	33	A	100.0								
														1.2	F	21.5	Y	77.3	H	34	H	57.1	Y	28.6	S	14.3				
																		100.0	C	35	C	100.0								

Abbreviations: A, alanine; C, cysteine; D, aspartic acid; E, glutamic acid; F, phenylalanine; G, glycine; H, histidine; I, isoleucine; K, lysine; L, leucine; M, methionine; N, asparagine; P, proline; Q, glutamine; R, arginine; S, serine; T, threonine; V, valine; Y, tyrosine. The colors indicate different percentage of amino acids. The lower the percentage of amino acid, lighter the color.

### 3.4. The env-c2v3 evolutionary distance of different tropism In CRF07_BC and CRF01_AE

**Figure 3:**
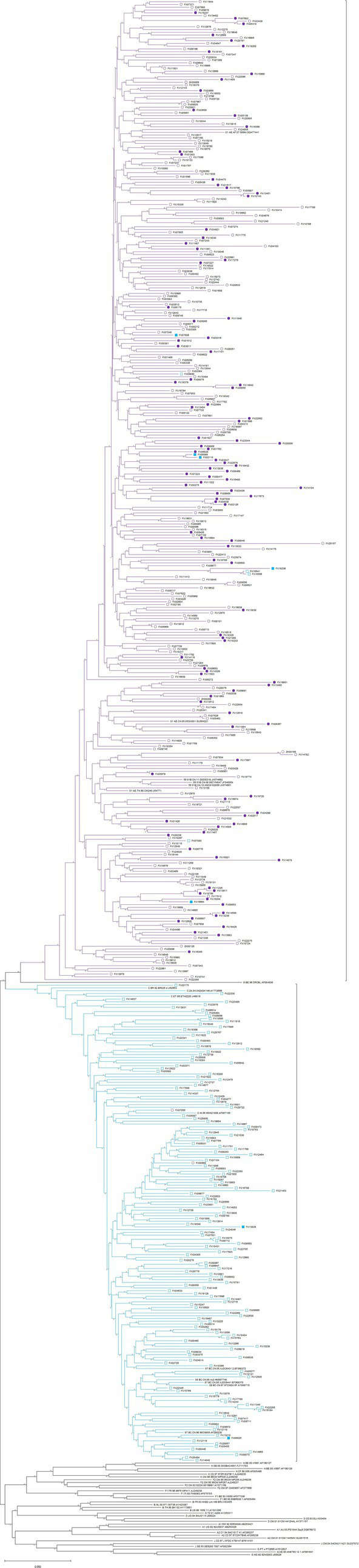
Phylogenetic tree (for HIV-1 env-c2v3 analysis) of the X4-tropic virus in CRF01_AE (the solid and hollow violet circles, respectively, represent X4-tropic and R5-tropic viruses) and CRF07_BC (the solid and hollow cyan squares, respectively, represent X4-tropic and R5-tropic viruses).

All env-c2v3 sequences of CRF01_AE and CRF07_BC were got together to construct a phylogenetic tree (for HIV-1 env-c2v3 analysis) by neighbor-joining and 1,000 bootstraps in the Kimura 2-parameter model. Figure 3 illustrates that 5 of 7 X4-tropic viruses in CRF07_BC were clustered within CRF01_AE rather than most CRF07_BC. We subsequently calculated the env-c2v3 evolutionary distance between tropism groups in CRF01_AE and CRF07_BC (Table 5). The evolutionary distance between X4-CRF07_BC and CRF01_AE (0.20559±0.01614) was closer (*p* < 0.0001) than the distance between X4-CRF07_BC and R5-CRF07_BC (0.26131±0.02228) (Supplementary Table 2).

**Table 5:** Estimates of env-c2v3 evolutionary distance between the groups.

Group 1	Group 2	Distance	Std. Err	*p*-Value
X4-CRF07_BC	R5-CRF07_BC	0.26131	0.02228	<0.0001
X4-CRF07_BC	CRF01_AE	0.20559	0.01614	
X4-CRF07_BC	R5-CRF01_AE	0.20195	0.01534	
X4-CRF07_BC	X4-CRF01_AE	0.21292	0.01571	
R5-CRF01_AE	X4-CRF01_AE	0.16561	0.01198	
R5-CRF07_BC	CRF01_AE	0.29697	0.02692	
R5-CRF07_BC	R5-CRF01_AE	0.29086	0.02631	
R5-CRF07_BC	X4-CRF01_AE	0.30926	0.02685	

The number of base substitutions and standard error estimate per site from between sequences are shown. Analyses were conducted using the Kimura 2-parameter model. All ambiguous positions were removed for each sequence pair (pairwise deletion option). Evolutionary analyses were conducted in MEGA11.

### 3.5. HIV-1 tropism switch

HIV-1 tropism switch was identified in 6 cases who received HAART (Supplementary Figure 3). The evolutionary distance within an individual is shown in Table 6. Five cases received HAART for more than 6 months except for one person named “LGS,” who had two-time points including FX06228 (108 days of HAART) and FX12668 (1,120 days of HAART). T1 was the earliest time point, and T3 was the latest time point. Table 6 indicates that HIV-1 tropism could change despite the individual receiving HAART.

**Table 6:** Estimates of env-c2v3 evolutionary distance within individual sequences.

PLWH	HIV-1 tropism & timepoint	Distance	
HXL		HXL-R5-07_BC (T3_FX18598)	HXL-R5-07_BC (T2_FX16541)
	HXL-R5-07_BC (T3_FX18598)		
	HXL-R5-07_BC (T2_FX16541)	0.00334 ± 0.00348	
	HXL-X4-07_BC (T1_FX10236)	0.07374 ± 0.01744	0.07000 ± 0.01697
ZZP		ZZP-X4-01_AE (T3_FX07602)	ZZP-X4-01_AE (T2_FX05310)
	ZZP-X4-01_AE (T3_FX07602)		
	ZZP-X4-01_AE (T2_FX05310)	0.03507 ± 0.01232	
	ZZP-R5-01_AE (T1_FX03438)	0.03507 ± 0.01232	0.01010 ± 0.00587
LGS		LGS-R5-01_AE (T2_FX12668)	
	LGS-R5-01_AE (T2_FX12668)		
	LGS-X4-01_AE (T1_FX06228)	0.07944 ± 0.01901	
STD		STD-X4-01_AE (T2_FX18200)	
	STD-X4-01_AE (T2_FX18200)		
	STD-R5-01_AE (T1_FX04647)	0.08476 ± 0.01806	
WCE		WCE-R5-07_BC (T2_FX13210)	
	WCE-R5-07_BC (T2_FX13210)		
	WCE-X4-07_BC (T1_FX06025)	0.05944 ± 0.01418	
YJB		YJB-R5-01_AE (T2_FX08951)	
	YJB-R5-01_AE (T2_FX08951)		
	YJB-X4-01_AE (T1_FX05979)	0.04136 ± 0.01371	

The number of base substitutions and standard error estimate per site from between sequences are shown. Analyses were conducted using the Kimura 2-parameter model. All ambiguous positions were removed for each sequence pair (pairwise deletion option). Evolutionary analyses were conducted in MEGA11.

### 3.6. The baseline immune levels In different HIV-1 subtypes and tropism

**Figure 4:**
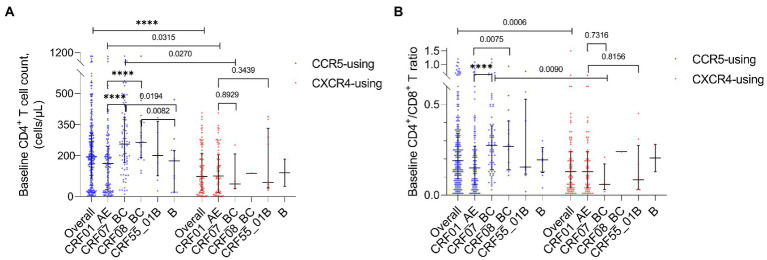
**(A)** Baseline CD4^+^ T cell count of all HIV-1 subtypes and overall tropism. **(B)** Baseline CD4^+^ T/CD8^+^ T ratio of all HIV-1 subtypes and overall tropism.

The baseline CD4^+^ T cell count (*p* < 0.0001) and baseline CD4^+^ T/CD8^+^ T ratio (*p* = 0.0006) of all HIV-1 R5-tropic infections were, respectively, higher than those of X4-tropic infections. For CRF07_BC, both baseline CD4^+^ T cell count (*p* = 0.0270) and baseline CD4^+^ T/CD8^+^ T ratio (*p* = 0.0090) were lower in the X4-tropic infection. For CRF01_AE, only the baseline CD4^+^ T cell count was lower (*p* = 0.0315) in the X4-tropic infection. Of all R5-tropic infections, the baseline CD4^+^ T cell count (*p* < 0.0001, *p* < 0.0001) and baseline CD4^+^ T/CD8^+^ T ratio (*p* < 0.0001, *p* = 0.0075) were lower in CRF01_AE compared to CRF07_BC and CRF08_BC (Figures 4A,B). The baseline CD4^+^ T cell count was lower in subtype B compared to CRF07_BC (*p* = 0.0194) and CRF08_BC (*p* = 0.0082) (Figure 4A). When the HIV-1 tropism was not considered, baseline CD4^+^ T cell count (*p* < 0.0001, *p* < 0.0001) and baseline CD4^+^ T/CD8^+^ T ratio were also lower in CRF01_AE (*p* < 0.0001, *p* = 0.0025) compared to CRF07_BC and CRF08_BC (Supplementary Figures 1A,B). The baseline CD4^+^ T cell count was lower in subtype B compared with CRF07_BC (*p* = 0.0115) and CRF08_BC (*p* = 0.0040) (Supplementary Figure 1A).

The baseline CD4^+^ T cell count of X4 HIV-1 in CRF01_AE was not significantly higher than that in CRF07_BC (*p* = 0.8929) and CRF55_01B (*p* = 0.3439). The baseline CD4^+^ T/CD8^+^ T ratio of X4 HIV-1 in CRF01_AE was not significantly higher than that in CRF07_BC (*p* = 0.7316) and CRF55_01B (*p* = 0.8156).

### 3.7. Factors associated with HIV-1 tropism

**Figure 5:**
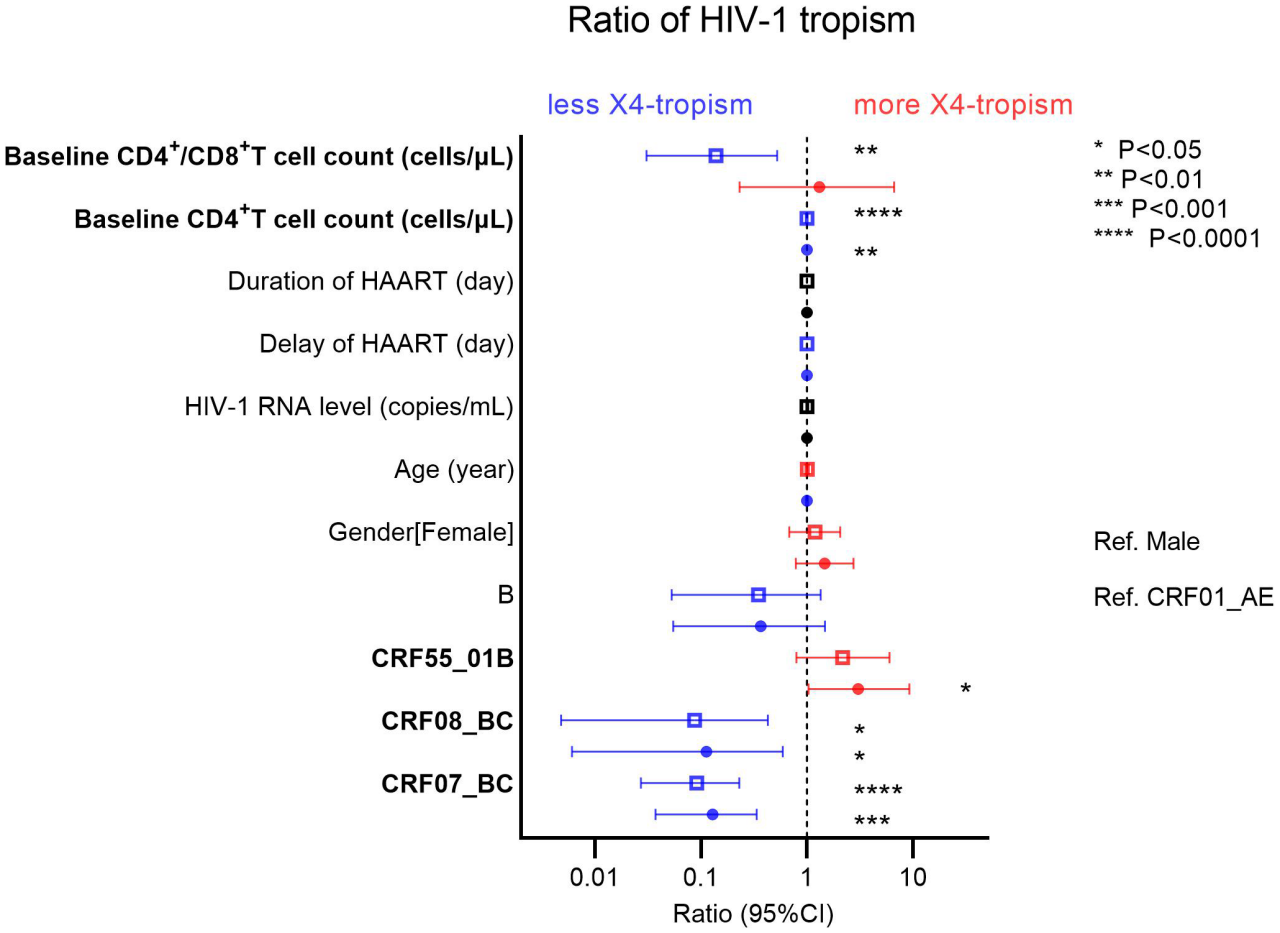
Univariate (hollow square) and multivariate (solid circle) logistic regressions of factors associated with HIV-1 tropism. The blue pattern represents the odds ratio of less than 1, while the red pattern represents the odds ratio of more than 1.

From the demographic characteristics of the study population, we performed univariate (the hollow square) and multivariate (the solid circle) logistic regressions for factors associated with HIV-1 tropism (Figure 5). The blue pattern represents the odds ratio of less than 1, while the red pattern represents the odds ratio of more than 1. As protective factors, a higher baseline CD4^+^ T cell count (odds ratio [OR] 0.9963, 95% confidence interval [CI] 0.9934–0.9990, *p* = 0.0097) was associated with less HIV-1 X4-tropism. Furthermore, taking CRF01_AE as a reference, CRF07_BC (OR 0.1283, 95% CI 0.03735–0.3353, *p* = 0.0002) and CRF08_BC (OR 0.1124, 95% CI 0.006087–0.5880, *p* = 0.0381) were associated with less HIV-1 X4-tropism. On the contrary, CRF55_01B (OR 3.035, 95% CI 1.0290–9.2040, *p* = 0.0443) was the risk factor relating to HIV-1 X4-tropism (Figure 5). In CRF01_AE, the baseline CD4^+^ T cell count was also the protective factor (OR 0.9956, 95% CI 0.9916–0.9991, *p* = 0.0217) for HIV-1 X4-tropism (Supplementary Figure 2).

### 3.8. Factors associated with continued CD4^+^ T cell count

**Figure 6:**
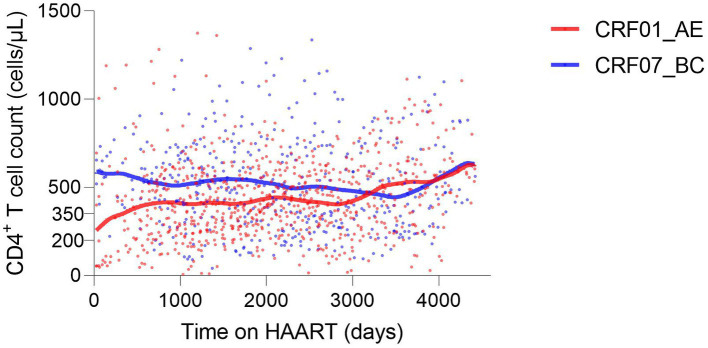
CD4^+^ T cell trajectories during HAART based on HIV-1 CRF01_AE and CRF07_BC.

Of 67 HIV-1 infections, considering factors associated with continued CD4^+^ T cell count, there were 41 infections with CRF01_AE and 26 infections with CRF07_BC. For a long time during HAART (from the initiation to the 3000th day of HAART), the CD4^+^ T cell count was higher in CRF07_BC, indicating that the overall immune level of infections with CRF01_AE was lower during early-stage HAART (Figure 6). The duration of HAART and the baseline CD4^+^ T cell count were protective factors (*p* < 0.0001), while age was a risk factor (*p* < 0.0001). CRF07_BC (*p* = 0.0137) and female sex (*p* < 0.0001) were protective factors compared with CRF01_AE and male sex, respectively. Additionally, the absent history of concurrent infection was also a protective factor (*p* = 0.0009) (Table 7).

**Table 7:** Factors associated with CD4^+^ T cell count during HAART.

Parameter estimates	Variable	Estimate	Standard error	95% CI (asymptotic)	|t|	*p* Value
β0	Intercept	198	27.32	144.4 to 251.6	7.246	<0.0001
β1	Age	−2.459	0.4973	−3.435 to-1.484	4.946	<0.0001
β2	Time on HAART (day)	0.07061	0.006192	0.05846 to 0.08276	11.4	<0.0001
	Reference[CRF01_AE]					
β3	HIV-1 subtype[CRF07_BC]	32.47	13.14	6.679 to 58.27	2.47	0.0137
	Reference[male]					
β4	Gender[female]	60.99	13.33	34.83 to 87.15	4.575	<0.0001
	Reference[yes]					
β5	History of concurrent infection[no]	44.47	13.35	18.26 to 70.67	3.33	0.0009
β6	Baseline CD4 T cell count (cells/μL)	0.7286	0.03248	0.6648 to 0.7923	22.43	<0.0001

## 4. Discussion

Studies have indicated that HIV-1 X4-tropism suggests a poor prognosis. Several HIV-1 subtypes, such as CRF01_AE, have a high proportion of X4-tropism, with which these are strongly associated. In a large population of treated Chinese living with HIV-1, virologic failure resulting from low-level viremia will cause a greater therapeutic burden. We need to find related factors of different HIV-1 tropisms and make efforts to reduce the occurrence of X4-tropic HIV-1. Herein, we retrospectively investigated HIV-1 tropism of low-level viral load HIV-1 infections in Guangdong, China.

Consistent with previous studies, we found a high prevalence of HIV-1 X4-tropism in CRF01_AE and CRF55_01B. There was a very low percentage of HIV-1 X4-tropism in CRF07_BC and CRF08_BC. Interestingly, the phylogenetic tree (for HIV-1 env-c2v3 analysis) and the evolutionary distance showed that CRF07_BC X4-tropic virus is closer to CRF01_AE in HIV-1 env-c2v3 rather than CRF07_BC R5-tropic virus. These results indicate that X4-tropic HIV-1 probably has similar genetic characteristics despite different HIV-1 clades. Therefore, similar to the previous study focused on HIV-1 subtype C (Coetzer et al., 2011), we suppose that extreme genetic divergence is required for the HIV-1 tropism switch in CRF07_BC and CRF08_BC.

We displayed the composition of every amino acid of the gp120 V3 loop in prevalent recombinant HIV-1 strains in Guangdong, China. We found statistically significantly higher proportions of IIe8/Val8, Arg11/Lys11, and Arg18/His18/Lys18 in X4-tropic HIV-1 in CRF01_AE and CRF07_BC. At the amino acid position 11 of HIV-1 V3 loop, we found more positively charged arginine and lysine in the X4-tropic virus, which was consistent with the 11/25 rule. At the 18th amino acid, we also found more positively charged including arginine, lysine and histidine in the X4-tropic virus. At the 25th amino acid, statistical difference was only found between X4-tropic-CRF01_AE and R5-tropic-CRF01_AE, displaying a lower proportion of aspartic acid and a higher proportion of glutamic acid in X4-tropic-CRF01_AE. At last, we concluded that the conserved position was “CTRPXNNTRXSXXXGPGXXFYXTGXIIGDIRXAXC,” while the “X” indicated the heterogeneous amino acids.

As the infection progresses, the HIV-1 coreceptor usages may change (Connor et al., 1997; Saracino et al., 2009) under a selective pressure within a treated individual. We found 6 HIV-1 infections that underwent tropism switch after 6 months of HAART in our study, indicating that HIV-1 tropism at a single time point cannot represent all tropisms of the PLWH during the whole treatment process. Therefore, in our study, we only took the single time point in the logistic regression for analysis of factors associated with HIV-1 tropism.

Of all HIV-1 subtypes in our study, we found that the lower baseline CD4^+^ T cell count, CRF01_AE, and CRF55_01B were the factors associated with HIV-1 X4-tropism. Since the sample size can influence statistical regression, we additionally analyzed the same factors in CRF01_AE without other HIV-1 subtypes (Supplementary Figure 2). The results also indicated that the lower baseline CD4^+^ T cell count was significant. Moreover, our study revealed that CRF01_AE, age, and history of concurrent infection were adverse factors for the recovery of CD4^+^ T cell count during HAART.

Our study reveals benign characteristics including the overall higher baseline CD4^+^ T cell count and baseline CD4^+^ T/CD8^+^ T ratio, especially a very low proportion of X4-tropism in infections of CRF07_BC. It seems that HIV-1 strains in CRF07_BC display a mild virulence. However, we observed an overall higher HIV-1 RNA load and a tendency of lower baseline CD4^+^ T cell count and baseline CD4^+^ T/CD8^+^ T ratio in X4-tropic CRF07_BC, even if no significant difference possibly due to the small sample size (Figures 4A,B). Further to the latest research that indicated a enhanced transmissibility and decreased virulence of CRF07_BC in China (Cheng et al., 2022), we propose that we need more evidence on virulence of X4-tropic CRF07_BC.

There were several limitations of our retrospective study that require consideration. First, the sample size was not in equilibrium because CRF55_01B, subtype B, and X4-tropic virus in CRF07_BC and CRF08_BC have a small sample capacity, which might have caused errors in statistical analysis. For example, we found no significant difference in X4-tropic HIV-1 regarding the baseline CD4^+^ T cell count (*p* = 0.8929) and baseline CD4^+^ T/CD8^+^ T ratio (*p* = 0.7316) between CRF01_AE and CRF07_BC. However, a previous study has provided compelling evidence that individuals infected with X4-tropic viruses had lower CD4^+^ T cell counts compared to those infected with R5-tropic viruses (Hu et al., 2022). Second, we had limitations in the sampling of multiple time points because these retrospective samples were previously collected by the Guangdong AIDS Diagnosis and Treatment Quality Control Center. For this reason, we did not analyze the continued changes in HIV-1 tropism, HIV-1 RNA, the resistance of the drug, most CD4^+^ T cell counts before and after HAART initiation, and even several phases of virologic responses. Moreover, the initial HAART regimen prescribed does not include integrase inhibitors and we did not analyze HIV-1 quasispecies in the peripheral blood. Lastly, we only used bioinformatics prediction for HIV-1 tropism based on HIV-1 env-c2v3 sequences with a lack of well-matched live virus isolates of the same categories for phenotypic testing. Further studies are needed to extensively explore and compensate for the limitations.

In summary, we provided an overall investigation of HIV-1 tropism and risk factors in different prevalent HIV-1 subtypes in 2014–2020 in Guangdong, China. Although we did not find precise factors associated with a very low proportion of X4-tropic HIV-1 in CRF07_BC and CRF08_BC, we showed the genetic similarity and difference of the HIV-1 V3 loop between CRF01_AE and CRF07_BC. Additionally, we supposed that extreme genetic divergence is required for tropism switch in CRF07_BC. This work may be helpful to fully explain the mechanism of HIV-1 coreceptor usage in further research. Our study indicated that lower baseline CD4^+^ T cell count contributes to HIV-1 X4-tropism. Therefore, we propose that the most effective way at present to reduce the occurrence of X4-tropic HIV-1 is pushing forward the progress of pre-exposure prophylaxis (PrEP) and early detection and treatment as far as possible to achieve a higher baseline CD4^+^ T cell count (Roul et al., 2018). Moreover, we have much to improve regarding the adherence and optimization of the HAART regimen to reduce “blip” and low-level HIV-1 viremia.

## Data availability statement

The data presented in the study are deposited in GenBank, accession number OQ603934-OQ604519.

## Ethics statement

The studies involving human participants were reviewed and approved by Institutional Review Board of the Guangzhou Eighth People’s Hospital (202033166). Written informed consent to participate in this study was provided by the participants’ legal guardian/next of kin.

## Author contributions

CZ conceived and designed the study, collected clinical information and performed experiments, analyzed data and wrote the manuscript. WC designed the study and revised the manuscript. YL performed experiments, and provided the experimental sample, demographic information, sequences of HIV-1 genotyping and analysis. LL provided professional comments and discussed the concepts of the manuscript. RH, YM, and JL assisted with manuscript preparation. All authors contributed to the article and approved the submitted version.

## Funding

This work was supported by Guangzhou Science and Technology Plan Project (202205110007) and Guangzhou Medical Key Discipline (Viral Infectious Diseases) from 2021 to 2023.

## Conflict of interest

The authors declare that the research was conducted in the absence of any commercial or financial relationships that could be construed as a potential conflict of interest.

## Publisher’s note

All claims expressed in this article are solely those of the authors and do not necessarily represent those of their affiliated organizations, or those of the publisher, the editors and the reviewers. Any product that may be evaluated in this article, or claim that may be made by its manufacturer, is not guaranteed or endorsed by the publisher.

## Footnotes

^1^ https://coreceptor.geno2pheno.org/index.php

^2^ https://tools.burnet.edu.au/phenoseq/

^3^ https://webs.iiitd.edu.in/raghava/hivcopred/submit.html

## Supplementary material

SUPPLEMENTAL TABLE 1A The composition of amino acids in CRF08_BC HIV-1 gp120 V3 loop.

SUPPLEMENTAL TABLE 1B The composition of amino acids in CRF55_01B HIV-1 gp120 V3 loop.

SUPPLEMENTAL TABLE 1C The composition of amino acids in subtype B HIV-1 gp120 V3 loop.

SUPPLEMENTAL TABLE 2 The statistical result of ordinary one-way ANOVA.

SUPPLEMENTAL TABLE 3 The mutiple timepoint of PLWH.

SUPPLEMENTAL FIGURE 1A Baseline CD4^+^ T cell count.

SUPPLEMENTAL FIGURE 1B Baseline CD4^+^ T/CD8^+^ T ratio.

SUPPLEMENTAL FIGURE 2 The ratio of tropism in HIV-1 CRF01_AE.

SUPPLEMENTAL FIGURE 3 Phylogenetic tree (for HIV-1 subtype confirming) of the 6 cases with HIV-1 tropism-switch.
