# Supplementary figures and images for: HIV-1 tropism in low-level viral load HIV-1 infections during HAART in Guangdong, China
Source: Front Microbiol. 2023 Apr 20;14:1159763. doi: 10.3389/fmicb.2023.1159763 (PMC10158941; doi:10.3389/fmicb.2023.1159763)

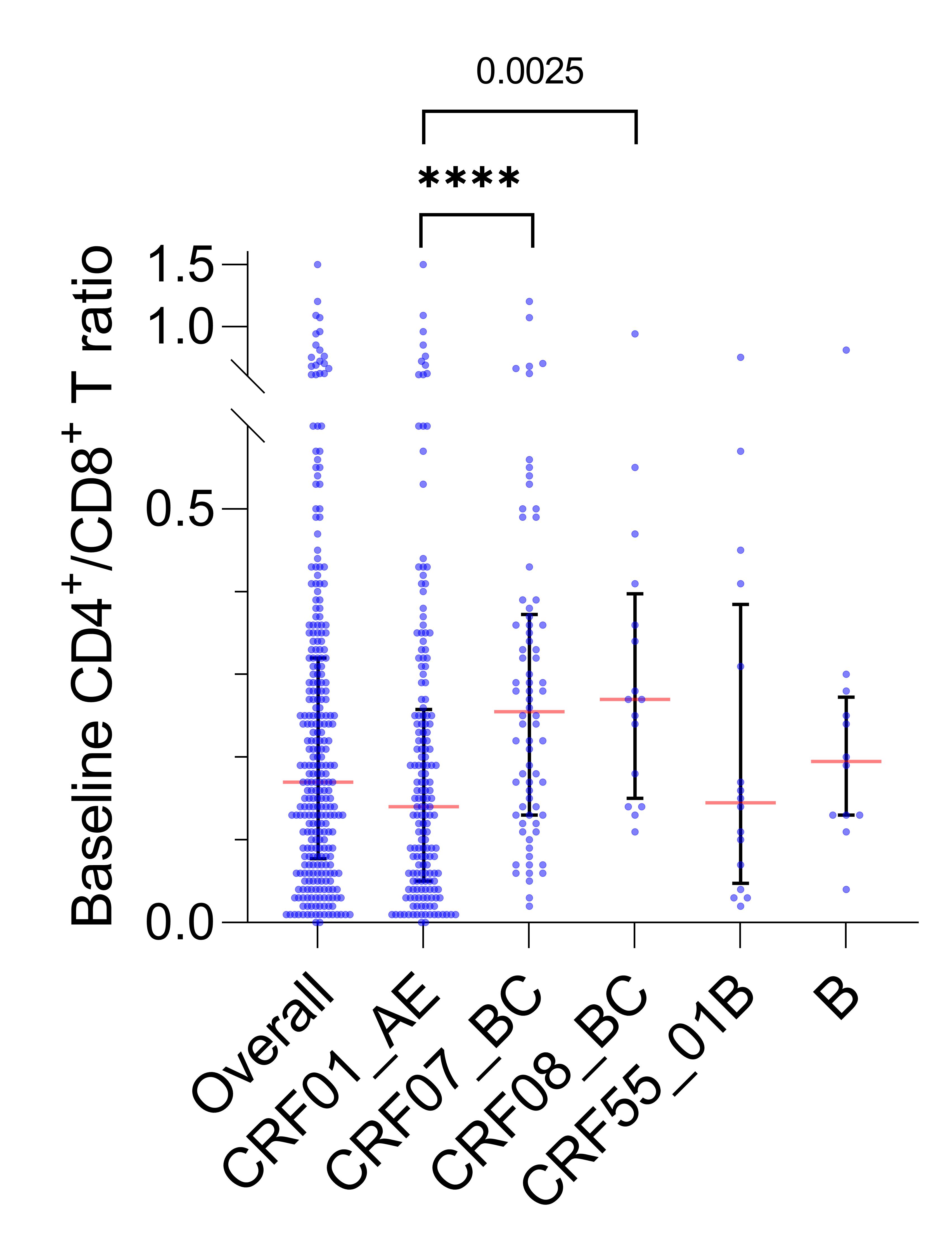

Supplement: SUPPLEMENTAL FIGURE 1A — Baseline CD4+ T cell count. [file Image_1.jpg]

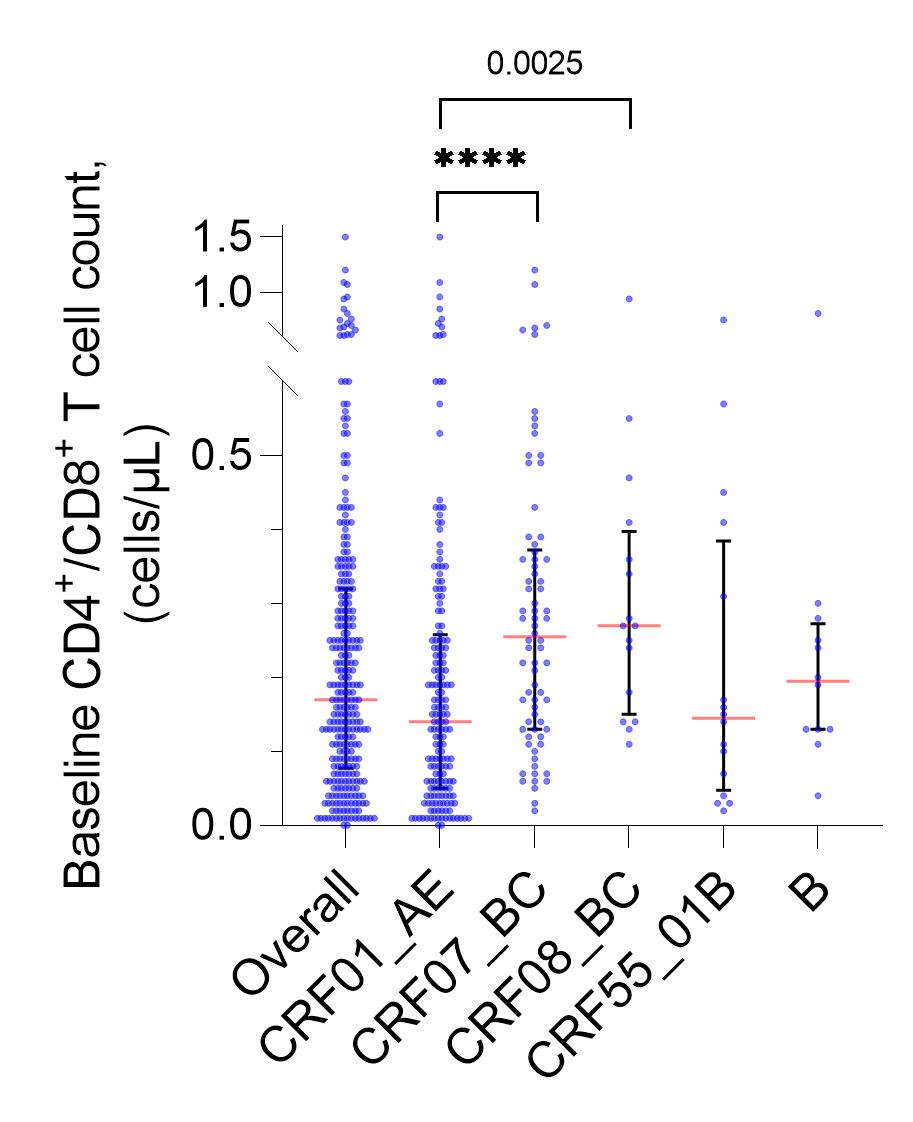

Supplement: SUPPLEMENTAL FIGURE 1B — Baseline CD4+ T/CD8+ T ratio. [file Image_2.JPEG]

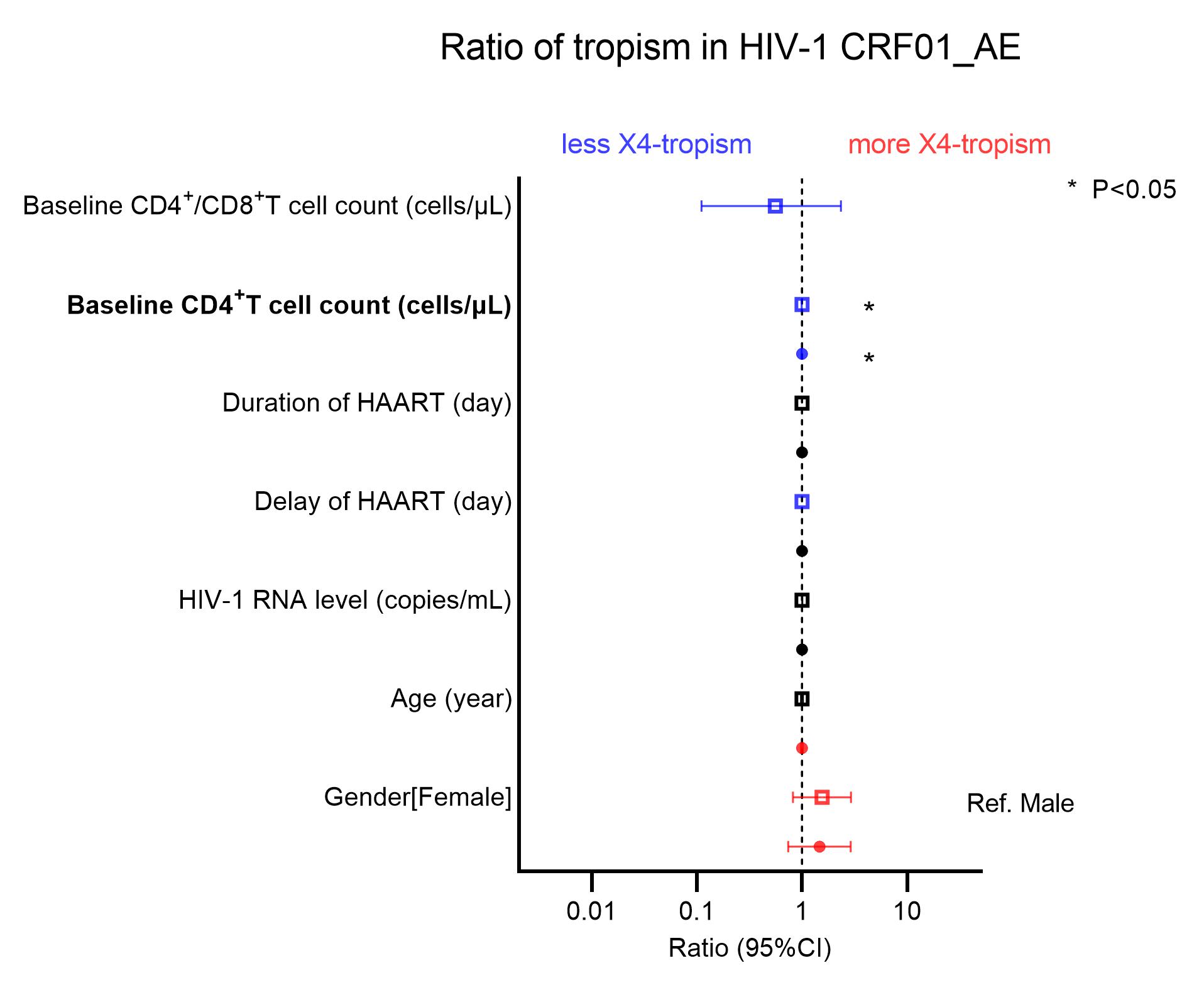

Supplement: SUPPLEMENTAL FIGURE 2 — The ratio of tropism in HIV-1 CRF01_AE. [file Image_3.JPEG]

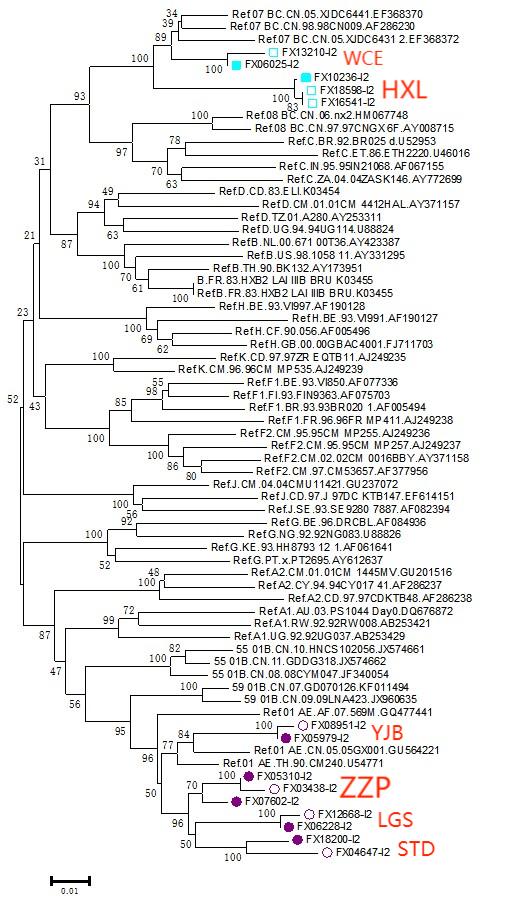

Supplement: SUPPLEMENTAL FIGURE 3 — Phylogenetic tree (for HIV-1 subtype confirming) of the 6 cases with HIV-1 tropism-switch. [file Image_4.JPEG]
